# Editorial for the Special Issue “MAPK in Health and Disease”

**DOI:** 10.3390/ijms25126663

**Published:** 2024-06-17

**Authors:** Francisco Centeno

**Affiliations:** Departamento de Bioquímica y Biología Molecular y Genética, Facultad de Ciencias, Universidad de Extremadura, 06006 Badajoz, Spain; pacenten@unex.es

The objective of this Special Issue was to collate recent advances in the understanding of MAPKs’ functions, particularly their roles in various pathologies, which constitute one of the most dynamic areas in cell signaling research. This compilation consists of five original research articles and one review, shedding light on the multifaceted roles of MAPKs.

Two of the published articles explored the interactions between MAPKs and commonly used drugs, elucidating previously unestablished connections.

In the first study [1], the researchers investigated potential drugs that have already been approved for clinical use that could target JNK3, a MAPK that is predominantly expressed in the central nervous system and is implicated in various neurodegenerative and neuroinflammatory conditions. Their findings revealed that sonidegib, known as an antagonist of the smoothened receptor (SMO) used in the treatment of certain medulloblastomas and basal cell carcinomas, binds with high affinity to JNK3’s active site at the nucleotide binding site (ATP). Using a microglia cell model treated with LPS, they demonstrated that sonidegib inhibits JNK-mediated signaling, subsequently reducing the expression of pro-inflammatory effectors and the migration of microglia cells. These results suggest sonidegib as a promising pharmacological treatment for inflammatory processes in the central nervous system.

In the second study [2], the researchers investigated the mechanisms underlying norketamine-induced damage in subepithelial and urinary epithelial cells. Norketamine, a metabolite of ketamine, demonstrated significantly higher cytotoxicity compared to its parent compound, ketamine, in human bladder epithelial cells. This cytotoxic effect was found to be apoptotic and dependent on endoplasmic reticulum stress activation, with MAPK ERK1/2 playing a crucial role mediated by intracellular calcium influx. These findings provide insights into ketamine-induced toxicity in urinary epithelia.

Further exploring the role of ERK1/2 MAPKs, this Special Issue highlights their significance in cerebral ischemia [3]. Studies in mice overexpressing ERK2 revealed exacerbated ischemic damage, while the inhibition of ERK1/2 activation attenuated such damage, particularly in inflammatory processes, underscoring the importance of ERK1/2 signaling pathways in ischemic stroke.

Another study delves into the prognostic implications of ERK5 in various cancers, including lung, prostate, and renal clear-cell carcinomas [4]. A higher ERK5 expression was correlated with a poorer prognosis in renal carcinoma patients. By elucidating the regulatory mechanisms involving miRNA-143, this study identifies ERK5 as a potential therapeutic target for kidney cancer treatment, highlighting its role in tumor cell viability, apoptosis, and angiogenesis.

Additionally, this Special Issue presents a novel degradation assay to identify new targets of FBXW7 [5], linking p38α to the phosphorylation-dependent degradation of JAZF1, a transcription factor that is implicated in endometrial stromal sarcoma.

The only review in this Special Issue focuses on the role of ERK1/2 MAPKs in tissue and organ regeneration processes [6]. Highlighting their central role in organ regeneration post-injury across various species, the review underscores the spatial–temporal dynamics of ERK1/2 activation and explores their potential applications in tissue therapy and regenerative medicine.

In summary, this Special Issue provides insights into the latest advances in MAPK research and their implications for health and disease, underscoring the dynamic nature of this field of study.
